# Impact of homeostatic body hydration status, evaluated by hemodynamic measures, on different pain sensitization paths to a chronic pain syndrome

**DOI:** 10.1038/s41598-024-52419-3

**Published:** 2024-01-22

**Authors:** Dmitry M. Davydov, Pablo de la Coba, Ana M. Contreras-Merino, Gustavo A. Reyes del Paso

**Affiliations:** 1 María Zambrano Senior Scholar, University of Jaén, Campus Las Lagunillas s/n, 23071 Jaén, Spain; 2 Laboratory of Neuroimmunopathology, Institute of General Pathology and Pathophysiology, Russian Academy of Sciences, Moscow, Russia; 3 Department of Psychology, University of Extremadura, Badajoz, Spain; 4 Department of Psychology, University of Córdoba, Córdoba, Spain; 5 Department of Psychology, University of Jaén, Jaén, Spain

**Keywords:** Psychophysics, Electrophysiology, Biomarkers, Hypertension, Fibromyalgia, Comorbidities

## Abstract

Contrasting findings on the mechanisms of chronic pain and hypertension development render the current conventional evidence of a negative relationship between blood pressure (BP) and pain severity insufficient for developing personalized treatments. In this interdisciplinary study, patients with fibromyalgia (FM) exhibiting clinically normal or elevated BP, alongside healthy participants were assessed. Different pain sensitization responses were evaluated using a dynamic 'slowly repeated evoked pain' (SREP) measure, as well as static pain pressure threshold and tolerance measures. Cardiovascular responses to clino-orthostatic (lying-standing) challenges were also examined as acute re- and de-hydration events, challenging cardiovascular and cerebrovascular homeostasis. These challenges involve compensating effects from various cardiac preload or afterload mechanisms associated with different homeostatic body hydration statuses. Additionally, hair cortisol concentration was considered as a factor with an impact on chronic hydration statuses. Pain windup (SREP) and lower pain threshold in FM patients were found to be related to BP rise during clinostatic (lying) rehydration or orthostatic (standing) dehydration events, respectively. These events were determined by acute systemic vasoconstriction (i.e., cardiac afterload response) overcompensating for clinostatic or orthostatic cardiac preload under-responses (low cardiac output or stroke volume). Lower pain tolerance was associated with tonic blood pressure reduction, determined by permanent hypovolemia (low stroke volume) decompensated by permanent systemic vasodilation. In conclusion, the body hydration status profiles assessed by (re)activity of systemic vascular resistance and effective blood volume-related measures can help predict the risk and intensity of different pain sensitization components in chronic pain syndrome, facilitating a more personalized management approach.

## Introduction

A handful of studies on pain control mechanisms have revealed their close relationship to blood pressure (BP) regulation. In certain patients with chronic pain syndromes, BP elevation or hypertension has demonstrated analgesic effects, coined as a 'pain adaptation' mechanism (BP-related hypoalgesia)^1,2^. Conversely, in other patients, hypertension has been linked to altered relationships between BP and acute pain sensitivity, assessed by measures such as pain threshold or temporal summation/wind-up (hyperalgesia-related BP elevation)^1,2^. However, both previous and current clinical guidelines for chronic pain management typically mention hemodynamic issues as mere side-effects of certain pain-killing drugs, like non-steroidal anti-inflammatory drugs (NSAIDs). These guidelines often overlook the hypertensive 'side-effect' associated with the effectiveness of medications, as well as the potential negative impact of antihypertensive medications on pain control^3–5^. Such an approach to chronic pain management may be considered overly simplistic, particularly in the context of systemic or integrative physiology, for the prescription of personalized therapy and lifestyle recommendations^6^. Furthermore, a previous study suggested a heterogeneous concept of chronic pain development, demonstrating that different baroreflex-related profiles of BP regulation could predict the effectiveness of pain control through personalized mental and physical activities in daily life^7^.

A recent review supports the idea of a heterogeneous concept of chronic pain development and suggests distinguishing central (more nociplastic) and peripheral (more nociceptive) mechanisms of pain for more effective, personalized pain control^8^. These mechanisms prove to be sensitive to dissimilar diet recommendations, surgical operations, and pain-killing medications, such as mood-regulating drugs or pain-regulating analgesics, including opioids and anti-inflammatory drugs^8^. For instance, the severity of pain in chronic syndromes like fibromyalgia (FM) is proposed to be determined by the interaction of different central and peripheral processes modifying responsiveness to peripheral stimuli, leading to either noxious (hypo- or hyper-algesia) or non-noxious (hypoesthesia or allodynia) outcomes^9–12^. Some authors suggest that detecting these clinical mechanisms of pain sensitization development can be achieved by assessing different forms of psychophysical sensitivity—either as a modified response to dynamic sensitization measures (specific responsiveness to temporal summation of fast or slow rates of the same intensity noxious stimulations) or to static sensitization measures, specifically pain threshold and pain tolerance^9,13–15^.

Previous studies have indicated that evoked pain sensitivity measures related to short static or persistent dynamic stimulations are similarly affected by high BP levels, although the effects differ between FM and healthy populations^16,17^. However, their specific relationship to two different forms of BP elevation—central (salt-sensitive with increased fluid volume, i.e., hypervolemic, hydraulic, or 'hyperkinetic') and peripheral (salt-resistant with reduced fluid volume, i.e., hypovolemic or 'vasoconstrictor')—has not been explored in humans to date^18–20^. Hypovolemic BP elevation, characterized by intermittent acute or prolonged systemic hypoperfusions, can impair cerebrovascular circulations by increasing cerebrovascular resistance as an autoregulation response. This is particularly relevant in older individuals and during regular body fluid redistributions in response to upright (e.g., during standing or orthostatic) positions, or other situations involving gravity, high altitude hypoxia, or fluid loss challenges. The coupling of hypovolemic or hypohydration conditions with such regular hypoperfusion events further establishes central conditioning as a mechanism of sensitization to peripheral stimuli^11,21–26^.

This hypovolemic form of BP elevation results from total blood volume contraction as a component of total body hypohydration. This condition also affects unstressed blood reserves diminishing the capacity to compensate for reduced venous blood return, ventricular filling, and cardiac output (CO). Consequently, it shifts the primary or predominant role from cardiac preload to cardiac afterload in supporting BP within the homeostatic range at rest and in response to hemodynamic challenges. Hypovolemic states pose a health risk, especially when coupled with occasional acute dehydration events, such as orthostatic (i.e., standing up) and other gravity-related challenges, which reduce stroke volume (SV)^25,27^. In these instances, BP elevation occurs due to a significantly increased afterload on the heart, determined by further enlarged systemic vasoconstriction as an overcompensating response to the lower reactivity of preload of the heart^25,27–29^.

In contrast, a hypervolemic form of BP elevation is a result of total blood volume expansion as a component of total body hyperhydration or fluid overload. This type of hypervolemic BP elevation, coupled with occasional acute rehydration events like a clinostatic challenge (i.e., lying down), is associated with blood reservoirs overflowing with unstressed blood volume. This occurs due to factors such as restricted compliance in capacitance vessels of the splanchnic region, leading to uncompensated high venous return, increased SV, and CO—indicating a homeostatic status with a permanent body hyperhydration state^30–32^. In addition to the overfull reservoirs of unstressed blood volume, restricted by maximal systemic vascular compliance, the body hyperhydration state is determined by the excessive deposition of sodium and water in the striated muscles, dermal interstitium/extracellular matrix, and lymphatics of the skin^33–36^. These compromised regulatory mechanisms, resulting in excessive water volume accumulations, may impair peripheral circulation and have an impact on peripheral conditioning and sensitization to stimuli applied to various organs and tissues, including the skin, intestines, muscles, and joints.

At present, the different hypo- and hypervolemic forms of BP elevation, as components of homeostatic body hydration statuses, cannot be reliably, promptly, and inexpensively assessed by instrumental methods in clinical and general populations. Common measures for assessing body hydration levels, such as total body water content and plasma/urine osmolality, are not ideal for indicating homeostatic hyper-, normo-, or hypo-hydration status in an individual. This is because they rely on population-derived ranges that cannot determine whether specific values of body hydration in individuals are within or outside their individual homeostatic ranges, as they do not assess compensating or regulating physiological responses to those hydration states^37^. In patients, medical histories, clinical features, and physical examinations, often with referral to specialists, along with the empiric ('trial and error') treatment method suggested by contemporary hypertension treatment guidelines, remain the standard for stratifying patients into these two different types of hypertension and determining their distinct care^38^. However, these methods are less suitable for individuals sampled from a general population^39^. Thus, the insufficiencies in traditional metabolic and clinical indicators of individually optimal body hydration states have been recognized as a problem in cardiovascular medicine^37,40^.

In this interdisciplinary study involving healthy participants and patients with fibromyalgia (FM), a chronic pain syndrome, the impacts of cardiac function (assessed by CO, a product of SV and heart rate) and total peripheral or systemic vascular resistance (SVR) on BP level regulation were explored as a methodology to indicate the two causal forms of its elevation associated with different homeostatic statuses of body hydration^38,41^. Cardiac output serves as an indicator of the effective blood volume stressed within the individual circulatory system, which is anatomically coupled with limited systemic vascular capacitance/compliance, determining fluctuations in finite unstressed blood volume reserves. This limitation of blood volume is particularly relevant in response to acute dehydration events challenging systemic hemodynamics, as seen in orthostatic stress. The cardiac preload mechanism is most relevant in eu- and hyper-hydration states (i.e., non-deficient water), differing only in the transfer function between stressed blood volume and unstressed blood volume reserves. However, during hypohydration states, the increase in cardiac afterload (i.e., total peripheral or systemic vascular resistance), serving as another main mechanism, compensates for deficits in unstressed blood volume reserves in response to gravity-related central dehydration challenges. This compensation regulates systemic hemodynamics and, consequently, BP.

In addition, glucocorticoid cortisol, sampled from hair, served as a secondary indicator of multiple interacting processes regulating water-electrolyte balance. Chronic cortisol levels are considered objective indicators of life stress experience, with effects on mood and the regulation of body arousal^42–44^. Furthermore, a long-lasting cortisol concentration was hypothesized to be associated with body fluid regulation mechanisms, influencing signals from high- and low-pressure arterial mechanosensitive receptors distributed in atrial, coronary, aortic, carotid, and pulmonary sites. These long-lasting cortisol effects are integrated with the chronic activities of antidiuretic hormone, aldosterone, and renin, influencing blood volume as a mediating component of BP regulation^45–49^. In contrast to blood, urine, and salivary cortisol samples obtained during, after, or shortly before a study, hair cortisol concentration (HCC) assesses its basal level. Therefore, it indicates cumulative (mal)adaptive stress- or arousal-related endocrine activity over relatively longer periods (several months) rather than current (mal)adaptive stress or arousal responses to occasional or acute challenges^50,51^. Thus, as one of the main factors determining the permanent body hydration status, the assessment of such persistent cortisol activity was considered particularly relevant for research aiming to assess the body hydration status on the date of a study.

In summary, this study was conducted on healthy participants and patients with FM, a disorder characterized by chronic widespread musculoskeletal pain^52,53^. Its aim was to explore the impact of central (hypervolemic or 'hydraulic') and peripheral (hypovolemic or 'vasoconstrictor') forms of BP elevation within the clinically normal and elevated range of BP, as defined by a recent national guideline^39^, on the so-called 'slowly repeated evoked pain' (SREP), pain threshold, and pain tolerance. These were considered independent dynamic and static components of FM risk. The identification of these two forms of BP elevation relied on measures of cardiac and systemic vascular functions, with their predominance in BP control closely related to the individual homeostatic status of body hydration as hyper- or hypo-hydration, respectively. Conceptualizing FM as a heterogeneous syndrome, its severity was associated with different forms of pain sensitization or hyperalgesia. It was hypothesized that all three forms of increased pain sensitization or hyperalgesia, represented by high SREP, low pain threshold, and low pain tolerance, would be associated with different blood volume-vasoconstrictor forms of BP level and BP reactivity control related to distinct homeostatic body hydration statuses.

## Results

A dynamic measure of pain sensitization (SREP) was not correlated with either static (pain threshold and tolerance) measures in both FM patients and healthy women (HW) (p > 0.20) while the static (pain threshold and tolerance) measures were moderately intercorrelated in the FM and HW groups (r = 0.50 and 0.49, respectively).

### Cardiac and vascular mechanisms determining the risk of FM mediated by different measures of pain

In the total sample, a mediation analysis indicated that clinostatic DBP rises predicted being in the FM group through a higher SREP (i.e., with an increased pain sensitization in the dynamic domain) if the rises were associated with a respective clinostatic SVR increase, i.e., with the ‘vasoconstrictor’ BP up-response to such a re-hydration event as transition from standing to lying posture: higher clinostatic DBP→ higher clinostatic SVR→ higher SREP→ being a patient with FM (B[bootstrap SE] =  − 0.074[0.050], bootstrap 95% CIs − 0.233 to − 0.009). The effect was found to be independent of pain threshold and tolerance (i.e., to the measures of pain sensitization in the static domain).

The same mediation analysis found that, irrespective of posture, tonic DBP reductions predicted being in the FM group through low pain tolerance and threshold (i.e., with an increased pain sensitization in the static domain) if they were coupled with tonically decreased SVR: lower DBP→ lower SVR→ lower pain tolerance and threshold→ being a patient with FM (B[bootstrap SE] = 0.019[0.016] and 0.036[0.021], bootstrap 95% CIs 0.001 to 0.060 and 0.010 to 0.093, respectively). Orthostatic SBP and DBP rises also predicted being in the FM group through an increased pain sensitization in the same static domain if they were coupled with orthostatic SV decrease: higher SBP and DBP→ lower SV→ lower pain threshold→ being a patient with FM (B[bootstrap SE] =  − 0.014[0.008] and − 0.021[0.012], bootstrap 95% CIs − 0.036 to − 0.004 and − 0.051 to − 0.006, respectively; the same results found for pain tolerance were not shown). The effects were found to be independent of SREP (i.e., the measure of pain sensitization in the dynamic domain).

An additional moderated mediation analysis showed that SBP and DBP elevations increased the risk of being in the FM group through a lower pain threshold (i.e., with an increased pain sensitization in the static domain) in those with higher HCC as an indicator of probable problems in body water regulation (> 178 pg/mg): higher SBP and DBP in those with high HCC→ lower pain threshold→ being a FM patient (B[bootstrap SE] =  − 0.023[0.015] and − 0.040[0.022], bootstrap 95% CIs − 0.066 to − 0.007 and − 0.098 to − 0.014).

### Impact of cardiac and vascular mechanisms regulating BP level on the severity of different types of sensitization to nociceptive stimuli in FM patients

**Figure 1:**
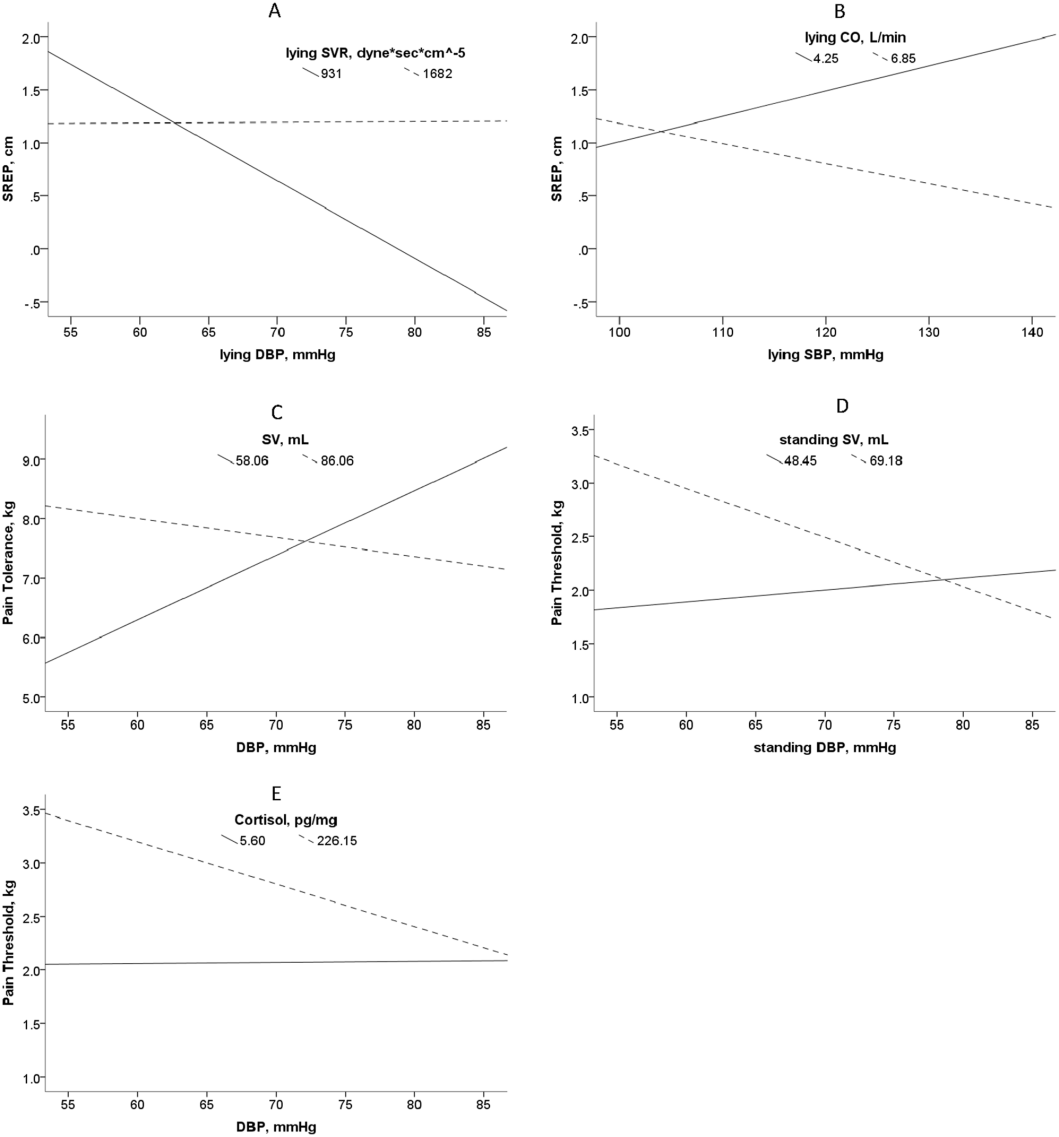
Interaction effects of lying diastolic blood pressure (DBP) and lying systemic vascular resistance (SVR) on ‘slowly repeated evoked pain’ (SREP) as a dynamic measure of pain sensitization (**A**). Interaction effects of lying systolic blood pressure (SBP) and lying cardiac output (CO) on ‘slowly repeated evoked pain’ (SREP) as a dynamic measure of pain sensitization (**B**). Interaction effects of tonic diastolic blood pressure (DBP) and tonic stroke volume (SV) on pain tolerance as a static measure of pain sensitization (**C**). Interaction effects of standing diastolic blood pressure (DBP) and standing stroke volume (SV) on pain threshold as a static measure of pain sensitization (**D**). Interaction effects of tonic diastolic blood pressure (DBP) and hair cortisol concentration (Cortisol) on pain threshold as a static measure of pain sensitization (**E**). As the surface stimulation area for eliciting pain was 1 cm^2﻿^, the scales of pain tolerance and pain threshold, expressed in kilograms (kg), are equivalent to kg/cm^2^.

According to the J-N technique, in FM patients, a higher reactive clinostatic DBP (> 70 mmHg) coupled with a lower clinostatic SVR (< 1400 dyne_*_sec_*_cm^-5^) as an effective compensating response to the central re-hydration event (i.e., transfer to lying from standing posture) predicted a lower SREP (i.e., a decreased pain sensitization in the dynamic domain) (B[Huber-White HC SE] = 0.099[0.029], t = 3.45, p < 0.0013; Fig. 1A). Another similar analysis showed that a higher reactive clinostatic SBP (> 135 mmHg) coupled with a lower clinostatic CO (< 3.6 L/min), as ineffective compensating central responses to the re-hydration event (i.e., transfer to lying from standing posture), predicted a higher SREP (i.e., an increased pain sensitization in the dynamic domain) (B[Huber-White HC SE] =  − 0.017[0.008], t =  − 2.17, p = 0.036; Fig. 1B). The effects were independent of pain thresholds and tolerances (i.e., the measures of pain sensitization in the static domain).

In the FM patients, a moderated mediation analysis showed that a lower reactive orthostatic DBP (< 60 mmHg) coupled with a higher orthostatic SV (> 86 mL) and lower SVR (< 2000 dyne*sec*cm^−5^), i.e., as an effective compensating response to a central de-hydration water redistribution event (the transfer from lying to standing posture), predicted a higher pain threshold (> 2.30 kg/cm^2^; i.e., a decreased pain sensitization in the static domain) (B[bootstrap SE] = 8.009[2.659], bootstrap 95% CIs 3.301 to 13.616). However, a lower orthostatic DBP (< 60 mmHg) coupled with a lower orthostatic SV (< 50 mL), as an uncompensated response to such a central de-hydration water redistribution event (transfer from lying to standing posture), predicted a lower pain threshold (i.e., an increased pain sensitization in the static domain) (B[Huber-White HC SE] =  − 0.028[0.014], t =  − 2.041, p = 0.048; Fig. 1C). Irrespective of posture, coupling of low tonic SV (< 62 mL) with low DBP (< 50 mmHg) or high DBP (> 95 mmHg), i.e., an uncompensated or compensated body hypo-hydration (water constriction) state, predicted a lower or higher pain tolerance (i.e., an increased or decreased pain sensitization in the static domain), respectively (B[Huber-White HC SE] = -0.005[0.002], t =  − 2.152, p = 0.037; Fig. 1D). The effects were independent of SREP (i.e., the measure of pain sensitization in the dynamic domain).

An additional moderation analysis showed that, irrespective of posture, low tonic DBP (< 62 mmHg) predicted decreased pain sensitization while high tonic DBP (> 100 mmHg) predicted increased pain sensitization in the static domain (i.e., a higher or lower pain threshold, respectively) in the FM patients with high HCC (> 220 pg/mg), i.e., in a compensated or uncompensated body water expansion state, respectively (B[Huber-White HC SE] =  − 0.024[0.009], t =  − 2.62, p = 0.012; Fig. 1E).

## Discussion

The findings of the present study confirm the main proposition that hemodynamic mechanisms related to blood volume, responding to acute or permanent body hydration challenges associated with temporal posture or general permanent water (im)balance, respectively, in attempts to maintain BP levels within a homeostatic range, could be associated with the risk and severity of fibromyalgia (FM) as a heterogeneous chronic pain syndrome. These mechanisms exert effects on different pain sensitization pathways. These findings align with other studies on FM patients and those with other chronic pain syndromes, indicating their heterogeneity and complicated etiopathogenesis^6,7,54,55^. The primary mechanism up-regulating BP levels in FM patients and contributing to two of the detected four paths to the disease was found to be associated with increased systemic vascular resistance. This manifested as non-optimal cardiac afterload responses to both clinostatic and orthostatic challenges (i.e., lying or standing postures), incorrectly compensating for acute changes in venous blood return to the heart and the respective cardiac preload responses to these challenges.

According to previous psychophysical pain studies, the effects observed in the pathways to pain chronicity/severity could be linked to the modification of pain sensation (resiliency/compensation or risk/aggravation mechanisms) at different neurophysiological levels, with partial or complete overlapping. These modifications may originate from the activity of first-order nociceptive neurons (involving a local negative feedback mechanism) and extend to the activity of second-order nociceptive neurons, with later peripheral 'wind-up' due to positive feedback mechanisms at the segmental spinal cord level, and further to third-order habituation-sensitization mechanisms, which either extend the 'wind-up' (resulting in hyperalgesia) or determine a 'wind-down' (resulting in hypoalgesia)^56–64^. The present study provides evidence of effects related to blood volume and vascular resistance on different pain resiliency/compensation and risk/aggravation mechanisms, involving multi-level interactions. The interaction of various metabolic, endocrine, and neurophysiologic mechanisms that control blood volume and vascular resistance reciprocally, in concordance with BP changes, can determine these effects. However, attributing these effects to a specific neurophysiological level of pain sensibility modifications, as well as establishing causality paths between body hydration status, BP levels, acute and chronic pain conditions, requires further research.

**Figure 2:**
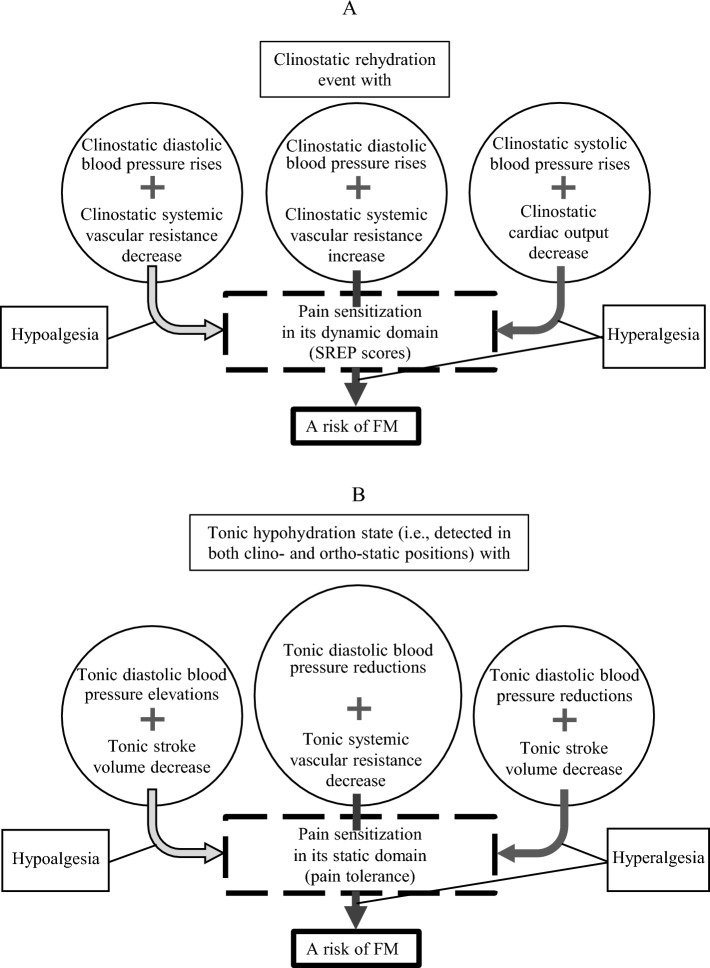
A schema of cardiovascular effects predicting fibromyalgia (FM) risk and severity of its sensitization component related to ‘slowly repeated evoked pain’ (SREP) as a dynamic measure of pain sensitization (**A**) and to pain tolerance as a static measure of pain sensitization (**B**).

For instance, the risk of FM associated with increased pain sensitization in its dynamic domain, as measured by high SREP scores, was found to be linked to clinostatic DBP rises along with a corresponding increase in systemic vascular resistance. This indicated an incorrect vascular emergency 'servo-control' mechanism compensating for water redistribution or hydraulic changes in response to a central rehydration event with increased venous blood return to the heart during the lying posture^65,66^ (see Fig. 2A). However, the severity of pain sensitization in this domain decreased (indicated by decreased SREP scores) in the FM group when clinostatic DBP rises were reciprocally coupled with a corresponding decrease in clinostatic systemic vascular resistance. This correct vascular 'servo-control' of BP changes dismantles the FM risk factor in responses to the rehydration event. In contrast, the severity of pain sensitization in the dynamic domain increased in the FM group if the main clinostatic cardiovascular pattern was accompanied by an additional rise in clinostatic SBP levels reciprocally coupled with a clinostatic cardiac output decrease. This represented an ineffective undercompensated cardiac preload response to the rehydration event, favoring cardiac afterload and accumulating the effect of the 'vasoconstrictor' form of BP rise. Such a predominantly 'vascular' mechanism regulating BP up-response to body water redistribution events, such as the lying position, may lead to habitually dysregulated cerebral and/or peripheral hypo-perfusions overcompensated by active cerebral and/or peripheral vasoconstriction^23,67–70^. This may contribute to the risk and higher severity of sensitization to pain in its dynamic domain^11,24,71^.

Unlike the dynamic pain sensitization domain, which is psychophysically assessed by a procedure like SREP, the static pain sensitization domain, measured by a psychophysical response like pain tolerance, was primarily associated with tonic (irrespective of posture) impairments in BP regulation linked to an uncompensated hypovolemic state (see Fig. 2B). In this scenario, a tonic reduction in DBP, determined by tonically reduced systemic vascular resistance in a reciprocal positive association, predicted FM risk with higher pain sensitization in this static domain measure (i.e., with low tolerance), independent of its dynamic domain. The higher severity of this static type of pain sensitization (i.e., lower pain tolerance) in FM patients was predicted by an additional positive coupling of tonically reduced DBP with tonically decreased stroke volume, serving as a physiological indicator of total body water content constriction^72^. The undercompensated 'vasodilator' BP down-response to hypovolemia, as a component of the permanent hypohydration state, may also induce cerebrovascular instability by hastening the decline in cerebral blood flow, particularly in specific brain areas such as the thalamus. This compromised regulation can further impact cerebral perfusion autoregulation, reducing cerebral oxygenation and the cerebral metabolic rate for oxygen, thereby contributing to increased fatigue, cognitive function decline, and heightened pain sensitization^11,25,70,71,73–75^. In contrast to this ineffective BP regulation, a negative coupling of tonically elevated DBP with tonically reduced stroke volume predicted decreased pain sensitization in the static domain (i.e., higher pain tolerance), independent of its dynamic domain.

**Figure 3:**
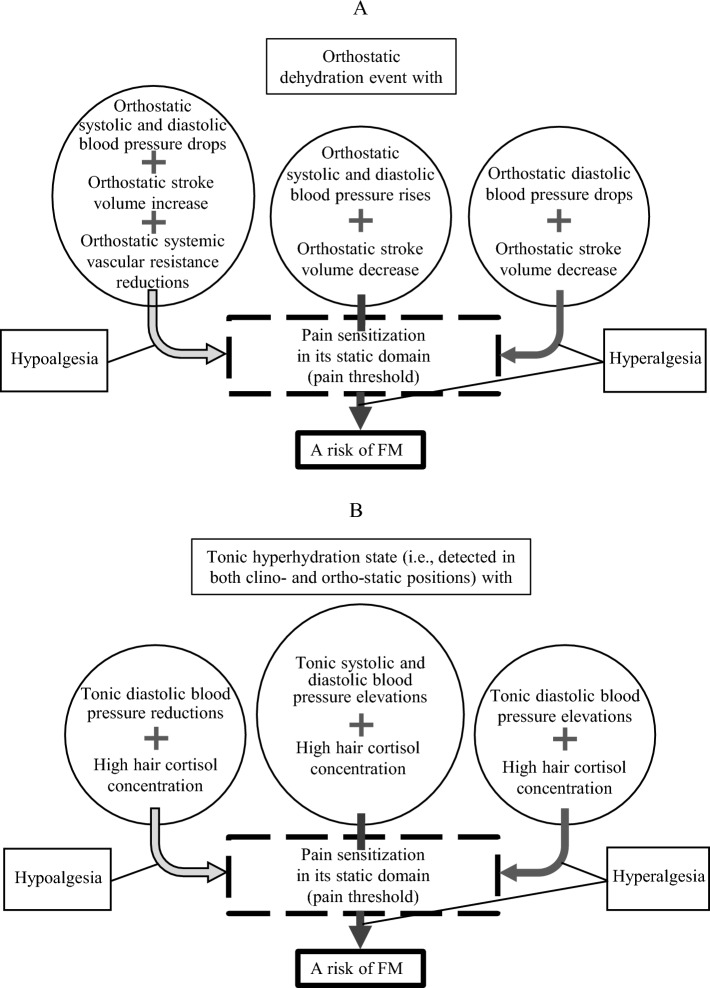
A schema of cardiovascular interaction effects (**A**) and cardiovascular and hair cortisol concentration interaction effects (**B**) predicting fibromyalgia (FM) risk and severity of its sensitization component related to pain threshold as a static measure of pain sensitization.

Another psychophysical measure of pain sensitization in the static domain, pain threshold, was primarily associated with impairments in the regulation of orthostatic reactivity of BP specifically in response to active standing (see Fig. 3A). In this case, the coupling of reactive rises in DBP (a proxy measure of the rise in systemic vascular resistance; see Supplementary Materials) with gravity-driven reductions in stroke volume, as a response to a water redistribution event with central de-hydration, also predicted a risk of FM with higher pain sensitization in this static domain measure (i.e., with low threshold), independent of its dynamic domain. Higher severity (i.e., a lower pain threshold) in the FM group was already associated with reactive orthostatic drops in DBP coupled with the same gravity-driven reductions in stroke volume, suggesting simultaneous dysfunctions in systemic vascular resistance and venous blood return, respectively, and a risk of hypoperfusion in multiple organs, including the brain^76^. In contrast, the coupling of orthostatic drops in DBP, indicative of systemic arterial vasodilation, with orthostatic stroke volume elevations compensating for these drops, was found to reduce severity in this static domain of pain sensitization (i.e., to increase pain threshold).

The reaction of BP in response to an acute dehydration event, such as active standing, might be mediated by atrial volume, high-pressure coronary, aortic, and carotid, as well as low-pressure pulmonary arterial mechanosensitive receptors that sense changes in central blood volume and arterial pressure^28,47^. However, incorrect interaction of these blood volume- and blood pressure-related mechanisms in response to regular dehydration events might impact multiple neurophysiologic, endocrine, and metabolic regulations in the central and peripheral activities of the brain, organs of the digestive system, muscles, and skin^23,25,45,47,49^. This, in turn, determines heterogeneous risks of chronic pain development with central and/or peripheral pain sensitization mechanisms, further impacting their severity^1^.

An additional mechanism predicting FM risk with higher pain sensitization in a static domain, and its severity in FM patients, as indicated by a lower pain threshold, was related to a positive coupling of tonically elevated SBP and DBP with a high, long-lasting cortisol level accumulated in hair. This cortisol level serves as an additional indicator of permanent body hyper-hydration, fluid overload, or a water expansion state (see Fig. 3B; also refer to Supplementary Materials). Increased BP, determined by a permanent hypervolemic or hyperhydration state uncompensated by a decrease in cardiac afterload (systemic vascular resistance), could lead to increased pain sensitization through several peripheral mechanisms. For example, impaired peripheral circulation with excessive vasodilation in peripheral tissues (in capacitance vessels with high compliance) such as the mesentery, muscles, and the skin—in response to their hyper-hydration—could lead to excessive water and sodium storage in the intestines, striated muscles, dermal extracellular matrix, and lymphatics of the skin, further sensitizing nociceptors to stimulations^30,33–36,45,46,77–79^. In contrast to the ineffective BP regulations during cortisol-driven fluid overload, a reciprocal coupling of tonic DBP reduction (a proxy indicator of the drop in systemic vascular resistance or decrease in cardiac afterload; see Supplementary Materials) with a high cortisol level—effectively compensated body hyper-hydration (water expansion)—predicted decreased pain sensitization in the static domain (i.e., a higher pain threshold), independent of its dynamic domain.

Thus, the dynamic domain of pain sensitization, identified as an FM risk factor, was associated with an over-compensated 'vasoconstrictor' DBP up-response to a rehydration event (i.e., transfer from standing to lying posture) with systemic vasodilation as its alleviating factor and a SBP rise against the background of cardiac output decrease as its aggravating factor. In contrast, static pain sensitization associated with low pain tolerance as an FM risk factor was linked to undercompensated tonic 'vasodilator' BP down-activity. Its severity worsened with additional coupling with a hypo-hydration state (permanently decreased stroke volume) and improved to lower severity by reversing tonic BP to up-activity coupled with a similar hypo-hydration state (permanently decreased stroke volume). Static pain sensitization with a low pain threshold as an FM risk factor was associated with a BP up-response to orthostatic events with central de-hydration (acutely decreased stroke volume), worsening in severity when reversing the orthostatic BP to down-response coupled with central de-hydration, and alleviation in severity by compensating the same orthostatic BP down-response with an increase in stroke volume. De- and re-hydration events only weakly differed in effects on cardiac (preload) components between healthy participants and patients in general, as indicated by clino-orthostatic stroke volume changes. However, vascular (afterload) effects were twice as reactive to them, with further mild effects on DBP changes in FM patients compared to healthy participants (see Supplementary Materials). As a separate mechanism, a hyperhydration state associated with a persistently high cortisol level and tonic BP elevation was a FM risk and severity aggravation factor through decreased pain threshold as a pathway to high pain sensitization. However, tonic BP reduction during this hyperhydration status was an alleviating factor for FM with high pain sensitization in this static domain.

Previous studies have suggested that the relationship between pain and BP can be complex and bi-directional^1^. The current findings contribute to further speculation about their relationships. The results demonstrate that the homeostatic body hydration status (i.e., hypo-, eu-, or hyper-hydration state) can act as a risk or resilience factor, directly or indirectly influencing acute pain sensitivity through effects on blood volume-vasoconstriction 'servo-control' of BP levels. This mechanism likely impacts pain sensitivity at different neurophysiological levels, as discussed earlier, guiding the organism towards or protecting it from pain chronification in response to occasional or regular nociceptive stimuli (e.g., as a high or low FM risk factor). Furthermore, individual body hydration statuses and their changes may be determined by a complex interaction of hereditary predispositions with living habits and environmental factors that epigenetically and conditionally modify the individual phenotype^80,81^. This includes effects from long-term exposure to specific conditions, such as some water-drinking and physical activity habits, dissociated or adaptively associated with climate, seasons, age, sex, (epi)genotype, and more, including pain experience as an occasional or regular event among others^81^. The Supplementary Materials provide examples related to aging and body mass effects on these processes. For instance, aging, considered as a proxy measure of metabolic activity and hydration decline, was found to affect BP regulation in the entire sample (similar effects were observed in both groups separately). These effects occurred through cardiac and vascular mechanisms, determining either elevated or reduced BP levels that were either compensated or uncompensated (by the systemic vasoconstriction/cardiac afterload mechanism) for hypohydration states. Body mass, taken as a measure of metabolic state related to the composition of fat and fat-free (water) body compartments, was also found to influence BP regulation in the overall sample through cardiac and vascular mechanisms. These mechanisms determined either elevated or reduced BP levels that were compensated or uncompensated (by cardiac preload and afterload mechanisms) for hypo- or hyper-hydration states. In individuals at high risk for pain chronification, the resulting homeostatic body hydration statuses and associated blood pressure regulating mechanisms may adaptively decrease severity or become maladaptive, increasing severity for living with chronic pain^1^.

The present findings offer a new perspective on pain control and management by considering medications and daily recommendations with specific effects on blood volume regulation as protective measures against pain chronification and additional remedies for pain severity of different origins. For instance, the use of NSAIDs could be personalized to enhance their pain-killing effectiveness, leveraging their 'side effect' on a renal retention mechanism compensating for hypovolemia, as identified in the present study^3,4^. Analogues of vasopressin, such as desmopressin, could be considered for use in patients with the hypovolemic type of pain development and aggravation, offering pain relief centrally (as AVPR1A agonists) and peripherally (through protecting the body from fluid loss via their antidiuretic and antihemorrhagic actions)^82–84^. Other proposed medications for individuals with hypervolemic pain aggravation could include those that reduce body sodium-volume content, such as mineralocorticoid receptor blockers, thiazide and thiazide-like diuretics, calcium channel blockers, α-adrenergic blockers, and nonspecific vasodilators^65^.

Real or simulated microgravity and hypergravity may also be explored to induce hyper- or hypo-volemia for respective compensating effects in patients with hypo- or hyper-volemic hyperalgesia^25^. Exposure to moderate (2000 m) or high altitude (3500 m) and their hypobaric equivalents for several weeks or days could be another approach for inducing blood volume expansion or contraction in patients with hypovolemic or hypervolemic hyperalgesia^46,85,86^. Medications that block or suppress plasma renin–angiotensin vasoconstrictor activity, such as angiotensin-converting enzyme inhibitors, angiotensin receptor blockers, direct renin inhibitors, β-adrenergic blockers, centrally acting α_2_-adrenergic receptor agonists, and reserpine, could be additional options in pain-killing treatment for patients with other hypovolemic types of pain aggravation^65,87^. This group of medications could be extended to drugs with mineralocorticoid and/or glucocorticoid activities, such as fludrocortisone, and non-drug therapies like bolus water drinking, due to their effects on increasing Na^+^ levels and therefore blood volume^76^.

In addition, a high salt diet (sodium or potassium feeding) could be recommended as an additional remedy for a hypovolemic type of hyperalgesia. This diet helps maintain and defend BP in response to challenges such as orthostatic stress and cerebral perfusion against brain ischemia associated with an acute ischemic stroke^67^. Conversely, a low salt diet renders the primary sodium-mediated mechanism inoperative, leading to its replacement with a renin-mediated vasculotoxic or vasoconstrictor mechanism that determines the risk of hypovolemic hypertension and a related type of hyperalgesia. This mechanism not only contributes to the development of hypovolemic hyperalgesia but also accelerates the progression of heart attack, heart failure, stroke, or kidney failure in the long term^67,88^. As a result, patients with chronic pain disorders like FM could be classified and treated based on their blood volume-related pain sensitization types, offering a more personalized approach in their management rather than relying solely on their specific diagnosis.

The present study delved into the concept that a rearrangement in the cardiovascular mechanisms supporting BP levels, specifically between predominantly cardiac preload or afterload, could serve as an indicator of the shift in the homeostatic status of body water content between hyper- and hypo-hydration states, respectively^30^. In this context, the assessment of the homeostatic body hydration status is attributed to the central nervous system (CNS) of the individual. The CNS integrates all relevant afferent signals from various central and peripheral receptors sensing the hydration state, initiating compensation or counteraction processes in response. Thus, this methodological approach is considered to be more reliable and personalized compared to the populational reference approach used in traditional methods for attributing body water content to hypo- or hyper-hydration status.

In summary, the evidence obtained in the present study confirms that chronic pain syndromes, such as fibromyalgia (FM), can manifest as heterogeneous diseases with diverse pain sensitization mechanisms. In certain FM patients, the pain sensitization mechanism may predominantly be associated with its dynamic component, such as high pain 'wind-up' to repeating stimulation. This mechanism can be predicted by BP rises coupled with cardiac afterload (systemic vascular resistance) increase and cardiac preload (cardiac activity) decrease in response to acute gravitation-related rehydration events. In other FM patients, the pain sensitization mechanism may predominantly be associated with its static components, such as low pain tolerance and pain threshold. These are predicted by tonic reductions in DBP and acute elevations in DBP, coupled with cardiac preload (cardiac activity) decreases that are either uncompensated or overcompensated by cardiac afterload (systemic vascular resistance). These responses occur in the context of chronic hypohydration states and acute gravitation-related dehydration events, respectively. A separate pain sensitization mechanism of chronic pain development associated with pain threshold as its static component was found to be related to the hypervolemic type of BP elevation. Other cardiovascular patterns regulating BP against permanent and acute challenges to body hydration and blood volume homeostasis predicted resilience mechanisms protecting against these types of pain sensitization mechanisms. These findings suggest that the control of optimal blood volume and body water balance can help prevent and control the risk and severity of chronic pain development related to homeostatic body hydration mechanisms (see some recommendations proposed for body hydration regulations above). Optimal water balance (mis)control could also determine or prevent the risk and severity of two volumetric or hydration types of essential hypertension development as comorbidities of pain disorders. These findings and related proposals warrant further investigation with more direct measures of central and peripheral water redistributions in response to chronic hydration states and acute hydration challenges, such as segmental and local (regional) bioimpedance measurement techniques, and similar investigations in a male sample and samples with other pain syndromes.

## Methods

The study was part of a larger project investigating pain processing in patients with FM with respect to cortisol and cardiovascular (CV) activity. However, none of the mean data of clino-orthostatic CV activity and their relationship to specific pain sensitization or hyperalgesia measures (SREP, pain threshold, and pain tolerance) have previously been reported.

### Participants

The patients were 48 women (aged 52.35 ± 8.95) diagnosed with FM by a rheumatologist according to the 1990 and 2010 American College of Rheumatology criteria^53^ and recruited via the Fibromyalgia Association of Jaén. The exclusion criteria were the presence of cardiovascular, neurological, inflammatory, metabolic, severe somatic (e.g., cancer) or psychiatric (e.g., psychotic condition or drug abuse) disorders and the use of medications affecting the cardiovascular system or the peripheral or central nervous system (excluding current FM-related medications). Current FM-related medications reported by patients and confirmed by medical records in this group included antidepressants (42 FM patients; 89.40% of the sample), anxiolytics (40 FM patients; 85.10% of the sample), non-opioids analgesics (39 FM patients; 83% of the sample), and opioids (26 FM patients; 55.31% of the sample). Thirty-seven healthy women (HW; aged 49.84 ± 6.65) comprised the control group. They met the same exclusion criteria as the patients and were also free from pain disorders and any medication use (except contraceptives). In the total sample, 43 participants were in the post-menopause period (18 FM patients and 25 HW), and 9 participants (5 FM patients and 4 HW) used oral contraceptives. As oral contraceptive use was found to be low in rates and equally distributed in both groups (10% in FM patients and 11% in healthy individuals), and it did not show confounding effects on the main findings, this factor was treated as random in all analyses. The participants´ clinical, psychological, and demographic data were obtained in a semi-structured interview along with self-report questionnaires. Table 1 shows the demographic (age, body mass index [BMI] assessed as weight/height^2^, and education in years), pain sensation, and cardiovascular data of both groups.

**Table 1:** Socio-demographic, pain sensation, and cardiovascular variables in fibromyalgia patients (FM) and healthy women.

Variables	FM patients (n = 48)	Healthy women (n = 37)	Bootstrap 95% CI		B (SE)	p
			Lower	Upper		
Age (years)	52.35 ± 8.95	49.84 ± 6.65	− 0.78	5.75	2.52 (1.69)	0.136
BMI (kg/m^2^)	28.12 ± 5.54	25.71 ± 3.22	0.63	4.32	2.41 (0.92)	0.008
Education (years)	11.33 ± 4.10	12.58 ± 4.10	− 3.01	0.46	− 1.25 (0.88)	0.161
HCC (Ln)*	4.10 ± 1.25	3.67 ± 1.18	− 0.12	0.93	0.43 (0.26)	0.121
SREP (cm)	1.11 ± 1.03	− 0.35 ± 1.20	1.02	1.95	1.46 (0.24)	0.001
Pain threshold (kg/cm^2^)	2.30 ± 1.38	3.67 ± 1.30	− 1.97	− 0.77	− 1.36 (0.30)	0.001
Pain tolerance (kg/cm^2^)	7.87 ± 3.11	9.87 ± 1.90	− 3.03	− 0.95	− 2.01 (0.55)	0.002
IBI (ms)	866.4 ± 109.8	895.6 ± 102.6	− 74.50	17.37	− 29.19 (22.79)	0.209
SBP (mmHg)	109.9 ± 14.7	106.7 ± 8.3	− 1.40	8.23	3.22 (2.50)	0.208
DBP (mmHg)	72.1 ± 10.3	70.5 ± 7.3	− 2.31	5.42	1.53 (1.93)	0.441
MAP (mmHg)	88.1 ± 11.8	85.9 ± 7.7	− 1.74	6.47	2.16 (2.12)	0.312
SV (ml)	71.7 ± 13.8	76.3 ± 16.7	− 11.47	2.11	− 4.59 (3.43)	0.188
CO (L/min)	4.98 ± 0.92	5.17 ± 1.27	− 0.72	0.30	− 0.19 (0.25)	0.469
SVR (dyne_*_sec_*_cm^−5^)	1461.8 ± 345.0	1388.0 ± 312.6	− 83.47	222.15	73.80 (74.58)	0.320

Confidence intervals (CI) and unstandardized beta (B) coefficients and standard errors (SE), and p from the regression analysis conducted with the bootstrap procedure.

*CI* confidence interval, *BMI* body mass index, *HCC* hair cortisol concentration, *IBI* interbeat interval, *SBP* systolic blood pressure, *DBP* diastolic blood pressure, *MAP* mean arterial pressure, *SV* stroke volume, *CO* cardiac output, *SVR* systemic vascular resistance, *SREP* slowly repeated evoked pain.

*Values of HCC in pg/mg were 112.70 ± 111.07 in FM and 76.45 ± 97.80 in healthy women.

### Measurement of hair cortisol concentrations

Samples of approximately 150 strands of hair were taken from the posterior vertex, cut as closely as possible to the scalp. The hair was wrapped in aluminum foil to protect it from humidity and light. For the analysis, 5-cm long hair strands (closest part to the scalp), equivalent to a 5-month period of hair growing, were separated from the obtained samples. Then, the hair was washed twice in isopropanol and, after drying, weighed and ground to break up its protein matrix. Cortisol from the interior of the hair shaft was extracted into gradient- or HPLC-grade methanol (for high-performance liquid chromatography) by incubation of the sample for 72 h at room temperature in the dark, with constant inversion using a rotator. After incubation, the samples were centrifuged, and the supernatant was evaporated until completely dry. Then, the extract was reconstituted in 150 µL of phosphate-buffered saline (PBS) at pH 8.0. The reconstituted sample was immediately frozen at – 20 °C for subsequent analysis. Finally, the hair cortisol concentration (HCC) of each sample was measured (in pg/mg) using the Cortisol Salivary ELISA kit (Alpco Diagnostics) with PBS at pH 8.0. More details on the method are available elsewhere^50^. The distribution of HCC was asymmetric, (right-skewed values), so the data were natural log-transformed to obtain a normal distribution.

### Cardiovascular recordings and data reduction

A Task Force Monitor (CNSystems, Graz, Austria) was used to record beat-to-beat cardiovascular variables. Two electrocardiograms (ECGs) were recorded and bandpass-filtered (0.08 to 150 Hz) by four electrodes applied to the chest, two close to the shoulders and two at the lower rib cage (Einthoven I and II). Four additional electrodes (two band electrodes at the xiphoidal level in the lateral chest, one band electrode at the lower nape, and a neutral electrode at the right ankle) were used for impedance cardiography (ICG) that was recorded using a 40 kHz current and filtered with a low pass (bandwidth 55.5 Hz) and a high pass (passband * 5 Hz) to remove artifacts and 50 Hz noise. Continuous blood pressure (BP) was taken from the first phalange of the second and third fingers of the right hand (positioned at the level of the heart) and oscillometric BP from the left brachial artery. The device recalibrates continuous finger BP according to brachial artery BP every 60 s, without interruption of recording. The ECG and ICG were sampled at 1000 Hz, and continuous finger BP at 200 Hz.

Inter-beat intervals (IBI, ms) and heart rate (HR, beats per min) were derived from ECG by detecting QRS complexes using an adaptive threshold decision algorithm implemented in the Task Force Monitor software and computing the time between R waves. Systolic, diastolic, and mean blood/arterial pressure levels (SBP, DBP, and MAP, mmHg) were obtained from the continuous finger BP recordings. Stroke volume (SV, ml) was obtained from ICG by using the Kubicek equation^89^; cardiac output (CO, L/min) was calculated as HR x SV; and total peripheral (TPR) or systemic vascular resistance (SVR, dyne_*_sec_*_cm^−5^) was computed by using the formula: 80 × (MAP—Central Venous Pressure)/CO^89^. These parameters were computed using the algorithms described by Wang et al. and Fortin et al.^90,91^, which reduced ventilation artifacts and motion noise, allowing for better correlations with the thermodilution technique compared to conventional methods^90^. Beat-to-beat data in all variables were inspected for artifacts. Detected artifacts were corrected by linear interpolation.

### Active clino-orthostatic (lying and standing) test

The “Chronic Pain Autonomic Stress Test” (CPAST) was used as a modified version of the active clino-orthostatic test previously adapted for chronic pain research^92^. The procedure included a 5-min baseline sitting position and a fixed postural test conducted in the following sequence: (1) 1 min of standing (first orthostatic phase); (2) 5 min of lying down (clinostatic phase); and (3) 5 min of standing (second orthostatic phase). The postural test was conducted twice, with a rest period of 20 min of sitting between sessions. Cardiovascular recordings started immediately after the respective postures were taken by the participant. Participants were asked to open their eyes while they were standing and during the baseline period and to close them while lying down.

### Pain induction and assessment

Pain sensation was evoked through a wireless pressure algometer, the Tracker Freedom (JTECH Medical, Lawndale, Salt Lake City, UT, USA), with a surface stimulation area of 1 cm^2^. The pressure algometer was applied manually guided by visual feedback on a computer screen. The algometer was inserted in a screw-piston specifically designed to fix and press the finger nails, allowing for reliable maintenance of stimulation pressure. Stimulation in the fingernails has been shown previously to accurately reflect overall pressure pain sensitivity^93^.

Participants were first instructed about the concepts of pain threshold (“when you first start to feel pain”) and tolerance (“when you reach the maximum stimulation pressure that you can tolerated”) as static tests and SREP (“evoked pain intensity assessment in response to a series of painful stimuli”) as a dynamic test. To assess the subjective intensity of the evoked pain during the SREP test, a 10 cm visual analogue scale (VAS) was completed following each pressure stimulus (“how intense was the pain?”). The anchors of the pain VAS were “painless” and “extremely painful”. Before the main test, VAS measurements were practiced through a training series of 6 stimuli of 5 s duration and different intensities in this order: 1.5, 2, 2.9, 1, 2.5, and 0.5 kg/cm^2^. The second finger nail of the left hand was used for this practice. After the practice or training phase and a resting period of 10 min, static evoked pain measures (threshold and tolerance) were obtained using the third left finger nail. Pain threshold and tolerance (in kg/cm^2^) were assessed at a rate of pressure increase of 1 kg/s pressure. After a rest period of 10 min, the SREP protocol was performed, delivering a single series of nine trials of pain stimulation (additional SREP details are provided in Ref.^94^). The intensity of pressure for SREP stimuli was calculated individually according to the formula: Intensity = Threshold + 1.25*(DF/4); where DF = Tolerance – Threshold^95^. This allowed for a low-to-moderate pain intensity in all participants. Pain stimulation for each trial in the series lasted 5 s during which pressure was kept constant. Five seconds after termination of each stimulus, the VAS pain measure was presented to evaluate pain intensity. Twenty seconds after each VAS assessment, the subsequent SREP trial was delivered, resulting in an inter-stimulus interval of 30 s. The difference in pain intensity (VAS ratings) between the 9th and the 1st trials was used to quantify SREP sensitization. Larger positive values indicate greater pain sensitization.

### Procedure

In the first part of the study, participants underwent an interview to ensure that they met the inclusion or exclusion criteria and to collect the clinical and self-report questionnaire data. During the second part of the study, the cardiovascular recordings were taken. After placing the BP transducers and electrodes, the CPAST was conducted. Averaged means of the cardiovascular variables for each of these periods were calculated and utilized in the present study. The analysis of fibromyalgia (FM) effects on rapid and slow second-by-second hemodynamic responses to clinostatic and orthostatic challenges is presented elsewhere. After the CPAST procedure with the cardiovascular recordings, BP measuring devices were removed, and the participants were engaged in the pain induction and assessment procedure as was described above. Hair samples were collected afterward. Participants were asked to refrain from caffeine, smoking, alcohol, and vigorous exercise for 3 h before the experiment and not to consume analgesic medications in the 24-h period prior to the evaluation. Written informed consent was obtained from all participants. The protocol of the research adhered to all relevant regulations and institutional policies, was performed in accordance with the Helsinki Declaration, and was approved by the Ethics Committee of the University of Jaén (Spain).

### Statistical analysis

The study was primarily powered on hair cortisol concentration (HCC) effects, as they were less explored and demonstrated a lower effect size previously compared to cardiovascular effects. The respective sample size was calculated based on an estimated effect size of 0.50 obtained from a previous meta-analysis of HCC effects^51^, with an error probability (alpha) of 0.05 and a beta error of 20%; thirty-five participants per group appeared optimal. Using means and standard deviations of cardiovascular responses to active standing from a previous study^96^, with an alpha of 0.05 and a beta error of 20%, a number of patients and healthy women < 23 in each group appeared optimal for all cardiovascular variables, including BP, SV, HR, CO, and SVR.

Three series of analyses were conducted for: (i) indicating cardiac and vascular mechanisms determining likelihood of being a patient with FM, i.e., the risk of getting FM as a chronic pain syndrome mediated by pain sensitizations with both dynamic (SREP) and static (pain threshold or tolerance) measures included in the same model as independent causal processes in the entire sample (patients and healthy participants); (ii) indicating the independent impact of blood volume-related central cardiac and peripheral vascular mechanisms regulating BP level on the intensity of both dynamic and static pain sensitization measures in patients with FM; and (iii) indicating impact of acute de- and re-hydration events (evoked by within-subject ortho- and clino-static challenges) and chronic hypo- and hyper-hydration states (assessed primary by between-subject difference in cardiac output, and secondary by between-subject difference in HCC) on blood volume-related cardiac and vascular mechanisms regulating BP level (see Supplementary Materials).

Regression analyses were used to test the main and alternative hypotheses by exploring mediation and moderation effects of cardiovascular variables (SBP, DBP, HR, SV, CO, and SVR) on measures of static (threshold and tolerance) and dynamic (SREP) sensitivities to painful stimuli, first, as parallel (independent) or serial paths in the risk of the disease development (FM vs. HW) and, second, as the parallel (independent) or serial mechanisms regulating intensity of these dynamic and static forms of pain sensitization within the FM group. The mediation models were explored using the SPSS macro 'PROCESS'^97^, as follows: (i) independent factor (e.g., DBP)→ mediation factor 1 (e.g., SVR)→ mediation factor 2 (e.g., CO or SV)→ mediation factors 3 (pain threshold or tolerance, and SREP included together in the same models to indicate their independence in the effects)→ dependent factor (FM vs HW), and (ii) independent factor (e.g., DBP)→ mediation factor 1 (e.g., SVR)→ mediation factor 2 (e.g., CO or SV)→ dependent factors 3 (pain threshold or tolerance, and SREP included together in the same models to indicate their independence in the effects) specifically in the FM group. The Johnson–Neyman (J-N) technique, included in the same SPSS macro, was used to detect regions of significant relationships in cases with significant moderation (interaction) effects. Since the study was cross-sectional, the results of the mediation and moderation analyses should be interpreted as associations, unless the obtained effects were explained by causal physiological mechanisms^1^.

The percentile bootstrap procedure (5000 bootstrap samples) was applied in the mediation analyses to generate non-parametric 95% confidence intervals (CIs) of regression coefficients to evaluate their significance. The observed relationships in the models were considered unlikely to be due to chance if the CIs did not include zero. The bootstrapping technique more accurately captures the shape of the sampling distribution and therefore has greater power to detect mediation effects^98^. Bootstrapping is often used as a flexible and robust nonparametric alternative to statistical inference based on parametric assumptions (such as a normal data distribution), because assumptions about distribution are not required by this method. The bootstrapping procedure allows assignment of measures of accuracy defined by CIs when the assumptions and stability of the results are uncertain, as is often the case in biological and psychological studies. Like randomization procedures, the bootstrapping procedure uses resampling techniques to construct a sampling distribution that can be used to make inferences about the population and to estimate CIs and statistics. The assumption of homogeneity of variance (homoscedasticity) may be violated in moderation models; to avoid overestimation of the goodness of fit of moderation models, the Huber-White (heteroscedasticity-consistent, HC) standard errors (SE) were used to determine the significance of the effects.

All parameter estimates are expressed as non-standardized (B) regression coefficients with standard errors (SE). Since all of the above-mentioned analyses were conducted to test specific hypotheses and followed the recommendations outlined by Rothman^99^, no adjustments for the number of inferences (e.g., correcting for multiple testing using the Bonferroni test) were made in either model. Two-tailed p-values < 0.05 were regarded as statistically significant in the moderation models. In those places where body positions were not explicitly mentioned, the effects were obtained for grand averaged values of cardiovascular measures.

### Supplementary Information

Supplementary Information.

## Data Availability

A dataset related to this manuscript may be found at https://osf.io/2bh35/. This dataset is not a part of this manuscript and has not undergone peer review.
