# Supplementary material for: Impact of homeostatic body hydration status, evaluated by hemodynamic measures, on different pain sensitization paths to a chronic pain syndrome
Source: Sci Rep. 2024 Jan 22;14:1908. doi: 10.1038/s41598-024-52419-3 (PMC10803325; doi:10.1038/s41598-024-52419-3)
Supplement: Supplementary file 1 — Supplementary Information. [file 41598_2024_52419_MOESM1_ESM.pdf]

**Supplementary materials**

**Title of related paper:** Impact of homeostatic body hydration status, evaluated by hemodynamic measures, on different pain sensitization paths to a chronic pain syndrome

**Subtitle of the supplementary materials:** Effects of de- and re-hydration events and hypo- and hyper-hydration states on cardiac and vascular mechanisms regulating BP level

**Authors:** Dmitry M. Davydov [a,b,*], Pablo de la Coba [c], Ana M. Contreras-Merino [d], Gustavo A. Reyes del Paso [e].

[a] María Zambrano senior scholar, University of Jaén, Jaén, Spain

[b] Laboratory of Neuroimmunopathology, Institute of General Pathology and Pathophysiology, Russian Academy of Sciences, Moscow, Russia.

[c] Department of Psychology, University of Extremadura, Badajoz, Spain

[d] Department of Psychology, University of Córdoba, Córdoba, Spain

[e] Department of Psychology, University of Jaén, Jaén, Spain

*** Corresponding author:**

Dmitry M. Davydov, University of Jaén, Campus Las Lagunillas s/n, 23071, Jaén, Spain; Email: d.m.davydov@gmail.com

Permanent link: https://doi.org/10.5281/zenodo.7796378

*Effects of de- and re-hydration events and hypo- and hyper-hydration states on cardiac and vascular mechanisms regulating BP level*

Orthostatic and clinostatic challenges as de- and re-hydration events were found to affect BP regulation through cardiac and vascular mechanisms: in response to standing from lying position, IBI decreased (Mean[SD] =-134[75] and 134[76] ms, t=-10.87 and -12.25, df=36 and 47, p<.0001), DBP increased (Mean[SD] =2.13[4.48] and 3.81[4.18] mmHg, t=2.89 and 6.33, df=36 and 47, p=.007 and <.0001), SBP remained unchanged (Mean[SD] =-1.27[5.09] and -0.65[5.60] mmHg, t=-1.52 and -0.81, df=36 and 47, p=.137 and .423), SVR increased (Mean[SD] =144[371] and 308[284] dyne∗sec∗$cm^{-5}$, t=2.366 and 7.51, df=36 and 47, p=.024 and <.0001), SV decreased (Mean[SD] =-21.92[21.77] and -26.73[16.47] ml, t=-6.13 and -11.25, df=36 and 47, p<.0001), and CO decreased (Mean[SD] =-0.77[1.43] and -1.11[1.01] L/min, t=-3.26 and -7.58, df=36 and 47, p=.002 and <.0001) in HW and FM groups, respectively. While de- and re-hydration events only weakly differed in effects on cardiac components between HW and FM groups as indicated by clino-orthostatic SV change (B[bootstrap SE] = -4.811[4.276], bootstrap 95% CIs = -13.201 to 3.882), vascular effects were twice more reactive to them as indicated in SVR responses (B[bootstrap SE] = 163.85[73.35], bootstrap 95% CIs = 20.270 to 313.310) with further mild effects on DBP increase (B[bootstrap SE] = 1.687[0.931], bootstrap 95% CIs = -0.178 to 3.428) in FM patients compared to HW.

Aging as a proxy measure of metabolic activity and hydration decline was found to affect BP regulation in the whole sample (the same effects were found in both groups, separately) through cardiac and vascular mechanisms determining different hydration states: (i) compensated hypohydration states (older age -> lower CO or SV -> higher

SVR -> higher SBP and DBP, e.g., for CO and SBP: B[bootstrap SE] = 0.696[0.174], bootstrap 95% CIs = 0.412 to 1.083 and older age -> higher SVR -> lower CO or SV -> lower SBP and DBP, e.g., for CO and SBP: B[bootstrap SE] = -0.393[0.086], bootstrap 95% CIs = -0.590 to -0.251) and (ii) uncompensated hypohydration states (older age -> lower CO or SV -> lower SBP and DBP, e.g., for CO and SBP: B[bootstrap SE] = -0.746[0.197], bootstrap 95% CIs = -1.188 to -0.418 and older age -> higher SVR -> higher SBP and DBP, e.g., for SBP: B[bootstrap SE] = 0.669[0.120], bootstrap 95% CIs = 0.453 to 0.924).

Body mass index (BMI) as a measure of metabolic state related to composition of fat and fat-free (water) body compartments was found to affect BP regulation in the whole sample through cardiac and vascular mechanisms determining different hydration states: (i) a compensated hypohydration (high fat) state (higher BMI -> higher SVR -> lower CO or SV -> lower SBP and DBP, e.g., for CO and SBP: B[bootstrap SE] = -0.214[0.091], bootstrap 95% CIs = -0.394 to -0.042), (ii) an uncompensated hyperhydration state (higher BMI -> higher CO or SV -> higher SBP and DBP, e.g., for CO and SBP: B[bootstrap SE] = 0.185[0.064], bootstrap 95% CIs = 0.070 to 0.325), and (iii) an uncompensated hypohydration (high fat) state (higher BMI -> higher SVR -> higher SBP and DBP, e.g., for SBP: B[bootstrap SE] = 0.332[0.143], bootstrap 95% CIs = 0.062 to 0.613).

A moderation analysis (B[Huber-White HC SE] = -0.0542[0.0250] and -0.0682[0.0299], t = -2.171 and -2.278, p = 0.033 and 0.025) confirmed that higher hair cortisol concentrations (HCC) as an indicator of hyper-hydration were related to SBP and DBP elevations as an uncompensated hyper-volemic effect (B[Huber-White HC

SE] = 0.0287[0.0090] and 0.0383[0.0151], t = 3.189 and 2.537, p = 0.002 and 0.013) in FM patients but not in HW (B[Huber-White HC SE] = -0.0255[0.0233] and -0.0299[0.0259], t = -1.094 and -1.157, p = 0.277 and 0.251). Another moderation analysis (B[Huber-White HC SE] = -2.011[0.783] and 0.451[0.237], t = -2.569 and 1.904, p = 0.012 and 0.061) showed that higher HCC as an indicator of hydration was related to lower SVR but higher CO in HW (B[Huber-White HC SE] = -1.247[0.588] and 0.392[0.131], t = -2.120 and 2.998, p = 0.037 and 0.004) but not in FM patients (B[Huber-White HC SE] = 0.764[0.517] and -0.059[0.198], t = 1.479 and -0.301, p = 0.143 and 0.765) as a hyper-volemic effect compensated by systemic vasodilation.
